# Larval spongy moth transcriptomic response to ingestion of broad-versus narrow-spectrum insecticidal *Chromobacterium* species

**DOI:** 10.1038/s41598-025-92113-6

**Published:** 2025-03-08

**Authors:** Michael E. Sparks, Sam D. Heraghty, Daniel Kuhar, Robert R. Farrar, Holly P. Bartholomew, Michael B. Blackburn, Dawn E. Gundersen-Rindal

**Affiliations:** USDA-ARS Invasive Insect Biocontrol and Behavior Laboratory, Beltsville, MD 20705 USA

**Keywords:** Forestry, Invasive species, Computational biology and bioinformatics, Microbiology

## Abstract

The PRAA4-1^T^ strain of *Chromobacterium subtsugae* was the first insecticidal bacterium to be registered by the U.S. Environmental Protection Agency for use in crop protection applications since approval for *Bacillus thuringiensis* was granted in 1961. *C. subtsugae*, a Gram-negative betaproteobacterium, exhibits oral toxicity against a broad range of important insects, including dipteran, coleopteran, lepidopteran, and at least some hemipteran and tetranychidan pests. *Chromobacterium sphagni* is a closely related bacterium exhibiting a distinctly narrower activity spectrum than that of *C. subtsugae*: it is toxic to lepidopteran, but not dipteran or coleopteran pest insects. The molecular mode of activity for either species is not well characterized at present, and it remains unclear whether these bacterial species affect insects similarly, notwithstanding their close evolutionary relatedness. In this study, synchronized third-instar larvae of the destructive lepidopteran forest pest, *Lymantria dispar dispar* (European spongy moth), were separately fed with cultures of *C. subtsugae* strain PRAA4-1^T^ or *C. sphagni* strain 14B-1^T^ and sampled after 24 h post infection. Gene expression levels in healthy reference versus treated insects were independently compared at the whole-insect and midgut-only tissue levels to characterize host-specific transcriptional responses to intoxication. Treatment induced up-regulation of such antimicrobial peptides as attacin and cecropin, of two cytochrome P450-encoding genes, and of gelsolin, a molecule involved in actin organization. Some differentially expressed genes were novel or uncharacterized, hence future work with lepidopteran species will be necessary to understand insect physiological responses to *Chromobacterium* infection.

**Supplementary Information:**

The online version contains supplementary material available at 10.1038/s41598-025-92113-6.

## Introduction

A number of bacteria within the genus *Chromobacterium* have been shown to impart deleterious effects on insects. *Chromobacterium subtsugae* has been identified as a potent and environmentally innocuous insecticidal bacterium, and was the first such agent registered for crop protection applications by the U.S. Environmental Protection Agency (EPA) since approval was granted for *Bacillus thuringiensis *(Bt) in 1961^1^. A commercial preparation of *C. subtsugae* is available for use as the organic insecticide Grandevo™ (Pro Farm Group, Davis, CA). An additional species in this genus, *C. sphagni*, has been observed in *Sphagnum *bogs in at least two states in the Eastern continental U.S., initially characterized due to its toxicity against lepidopteran insect larvae^2^.

The activity of *Chromobacterium* species can vary considerably against different orders of insects. When tested against representative Lepidoptera, Diptera, Coleoptera and Hemiptera, *C. subtsugae *exhibited toxicity against all four orders^3–5^. When tested against Lepidoptera, Diptera, and Coleoptera, *C. sphagni *was observed to be toxic only to Lepidoptera^6^, and *C. vaccinii *toxic only to Diptera^7^. These observations suggest the existence of several distinct modes of action. However, the multiple molecular mechanisms responsible for these modes are not yet well understood beyond the toxins described by Asolkar et al.^8^.

Genome sequencing and analyses have helped to elucidate some of the chromosomal genetic determinants presumably underlying *C. subtsugae*’s insecticidal properties^9,10^, and this insect toxicity appears entirely distinct from the plasmid-borne pesticidal proteins characteristic of Bts^11^. *C. subtsugae *has been described to produce three insecticidal factors, including “chromamide A”, violacein and one unidentified compound^8^. *C. subtsugae* also encodes a cyanide biosynthetic gene cluster, *hcnABC*, that may contribute to toxicity as reported for *Chromobacterium *sp. Panama^12^.

Although *C*. sp. Panama was isolated from the gut of a mosquito, and is toxic to mosquitos, other species have not been observed to be associated with insects, and it is unclear whether *Chromobacterium* species benefit from their toxicity to insects. Production of hydrogen cyanide has been demonstrated to contribute to the toxicity of *C*. sp. Panama^12^, but other factors contribute, as non-living cyanide-free preparations of the toxic bacteria can be formulated^13^. However, *C. sphagni* 14B-1^T^ also encodes a cyanide biosynthetic gene cluster and, although it exhibits similar oral toxicity to that of *C. subtsugae *against lepidopterans, it is not toxic to dipteran and coleopteran insects^6^. These observations suggest that, if cyanide is involved in toxicity, it likely operates as part of a polygenic, multifactorial system.

The European strain of spongy (formerly gypsy) moth, *Lymantria dispar dispar *(Lepidoptera: Erebidae), is a serious forest pest in the Northeastern United States and Canada. Spongy moth is a polyphagous insect, with North American larval populations feeding on over 300 different shrub and tree species, including forest, shade, ornamental and fruit trees and shrubs^14^. Here, synchronized third-instar larvae of *L. dispar dispar* were fed with cultures of either *C. subtsugae* strain PRAA4-1^T^ or *C. sphagni* strain 14B-1^T^ and examined transcriptionally 24 h post infection. Gene expression levels in healthy reference and treated insects were independently compared at the whole-insect and midgut-specific tissue levels to characterize host-specific transcriptional responses to ingestion of cultures in a species-specific manner with respect to bacterium.

A more complete representation of *L. dispar*’s transcriptomic response to ingestion of *C. subtsugae* or *C. sphagni *should help elucidate key toxicologic factors involved in the establishment and progression of pathology (if at least in a lepidopteran species) of broad- versus narrow-toxicity strains, and will provide an interesting basis for comparison with lepidopteran response to bacterial infection mediated by Bt (as has been reported elsewhere, e.g^15^), among other biotic challenges.

## Materials and methods

New Jersey standard strain spongy moth, *L. dispar dispar*, eggs were obtained from USDA APHIS, Otis Air National Guard Base, Buzzards Bay, MA. Using methods previously described^6^, larvae were hatched and reared on high wheat germ artificial diet^16^ in 180-ml plastic cups with paper lids under conditions of 24 ± 1 °C, RH 55–60% and L16:D8. Synchronized larvae were fed 300 µl lyophilized diet pellets^17^ that had been rehydrated with whole cultures of either *C. subtsugae* strain PRAA4-1^T^ or *C. sphagni* strain 14B-1^T^, or sterile water (control). Bacteria were cultured for 72 h in Luria-Bertani broth on an orbital shaker at 150 rpm and 25^o^ C. Larvae were maintained on the inoculated diet at room temperature for 24 h prior to preparing RNA. Whole-insect RNA preparations were made for one set of insects, and midguts were extracted from a distinct set. This resulted in two, non-orthogonal comparisons (whole-insect samples comprised midgut tissues and thus could not be considered entirely independent): (1) comparison of treated vs. control whole-insect samples and (2) comparison of treated vs. control midgut samples. Comparisons utilized a shared set of tissue-specific control insect measurements—that is, PRAA4-1- and 14B-1-treated midgut test samples were compared against the same negative control midgut samples, and PRAA4-1- and 14B-1-treated whole-insect samples were similarly compared against the same water control whole-insect samples. When considering the two tissue types assayed under each of the two insecticidal bacterial species, a total of four base-level comparisons was possible. Three biological replicates were prepared apiece for each of these six sample categories.

Midgut tissues (containing anterior, middle and posterior regions, as well as the peritrophic matrix) from control, *C. subtsugae* strain PRAA4-1^T^ fed, or *C. sphagni* strain 14B-1^T^ fed larvae, respectively, were dissected from the larvae at a time point after 24 h of feeding, rinsed thoroughly in 1x phosphate buffered saline (PBS) buffer to remove debris, and pooled (10 midguts per replicate). Midgut tissue RNAs were extracted immediately upon dissection and used to generate RNA-Seq libraries. Whole larvae from each treatment were prepared similarly (10 midguts per replicate). Whole larvae or midguts were placed in 2 mL matrix A lysing tubes and pulverized using MP BioMedicals’ (Solon, OH) Fastprep 24 for 60 s in 300 µl lysis buffer. RNA was extracted utilizing the RNAqueousTM Total RNA Isolation kit (ThermoFisher, Waltham, MA) protocol and further purified using Turbo DNAse (ThermoFisher, Waltham, MA) to remove any DNA contamination. RNA concentration was determined using Promega’s (Madison, WI) Quantas fluorometer. Samples were aliquoted and sent to the University of Georgia Genomics Facility (Athens, GA) for further processing, and libraries were run on an Illumina HiFi instrument. Total RNA-Seq sequencing volumes are presented in Table 1. A principal component analysis was conducted on these RNA-Seq data using the vst and plotPCA utilities included in DESeq2 (v1.36.0^18^). Reads from all 18 samples were combined and in silico normalized using utilities provided with the Trinity RNA-Seq assembler (v2.15.1^19^). Normalized reads were then aligned to an *L. dispar dispar *genome assembly—JAEVLZ010000000, available under GenBank accession identifier GCA_016802235.1^20^—using the STAR alignment program (v2.7.9a_2021-06–25^21^). Short read alignment results were merged and used to produce a genome-guided transcriptome assembly with Trinity.

**Table 1:** Raw sample-specific sequencing volumes. A target length of 150 bp was used for each paired read.

		Whole-Insect			Midgut		
		biorep 1	biorep 2	biorep 3	biorep 1	biorep 2	biorep 3
Control	Read pairs	52,193,939	13,321,780	13,496,207	11,099,265	37,793,999	45,731,814
	Bases	15,762,569,578	4,023,177,560	4,075,854,514	3,351,978,030	11,413,787,698	13,811,007,828
*C. subtsugae* (PRAA4-1^T^)	Read pairs	13,624,851	11,105,740	12,914,736	42,407,106	35,397,507	11,755,487
	Bases	4,114,705,002	3,353,933,480	3,900,250,272	12,806,946,012	10,690,047,114	3,550,157,074
*C. sphagni* (14B-1^T^)	Read pairs	36,636,450	15,373,584	12,084,644	9,644,628	16,893,942	13,508,348
	Bases	11,064,207,900	4,642,822,368	3,649,562,488	2,912,677,656	5,101,970,484	4,079,521,096

DESeq2, in conjunction with the salmon mapping tool (v1.9.0^22^), was utilized to identify differentially expressed genes (DEGs) with statistical significance in the four comparisons noted above: 14B-1 midgut vs. control midgut, 14B-1 whole-insect vs. control whole-insect, PRAA4-1 midgut vs. control midgut, and PRAA4-1 whole-insect vs. control whole-insect. A gene was designated as differentially expressed if it exhibited a false discovery rate of 0.05 or less and at least a doubling (or halving) of mean abundance (i.e., | log_2_(fold_change) | ≥ 1.0) between the sample types being compared. To obtain both gene- and transcript-level expression estimates, the RSEM expression estimation method (v1.3.3^23^), using read alignment results produced by bowtie 2 (v2.4.5^24^), was invoked by the align_and_estimate_abundance utility subsumed by the Trinity software package. Expression measurements were conveyed using the Transcripts per Million unit (TPM^25^). The ggVennDiagram R package^26^ was used to prepare a Venn diagram summarizing counts of DEGs shared among the four treated sample types (relative to expression measurements in their respective control samples).

Assembled transcripts were compared with the 18 July 2024 NCBI NR protein database using the BLASTx-like mode of DIAMOND (v2.0.4^27^) with default parameters. In addition, a longest open reading frame (ORF) was found for each mRNA sequence after translating in six frames using the transeq program from EMBOSS (v6.6.0.0^28^). Longest ORFs were then compared with the Pfam database (accessed 12 September 2023^29^) using HMMer (v3.3.4^30^) with default parameters. Associated Gene Ontology (GO) terms for protein family matches were identified using the pfam2go table (v2024-01–17^31^).

Biosynthetic gene cluster (BGC) composition of two bacterial genomes previously published by the authors—*C. subtsugae* PRAA 4-1^T^(GCA_001676875.1^9^) and *C. sphagni* 14B-1^T^(GCA_001855555.1^2^)—was analyzed here. BGCs were identified in each genome assembly using the bacterial antiSMASH program with relaxed settings (v7.1.0^32^) and then compared for shared identity.

## Results

The assembly consisted of 262,773,762 base pairs assembled into 326,665 transcripts, of which 323,089 were unique. A total of 111,414 assembled transcripts (i.e., 34.11% of total) exhibited one or more hits to reference protein sequences, with the remainder corresponding to transcripts not yet annotated in reference databases, transcripts whose sequences have diverged too greatly for homology to be detected by the alignment method, misassembled transcripts, or other possibilities. Protein family (Pfam) hits for inferred translation products were observed for 45,314 transcripts (13.87% of total), and of these, 12,054 (26.60%) could be linked with Gene Ontology information. These sequence annotations, as well as transcription expression measurements, are provided on the worksheet labeled “all_isoforms_with_TPM” of the supplementary file, “ExprAnnot.SupplementaryMaterial.xlsx”.

**Fig. 1:**
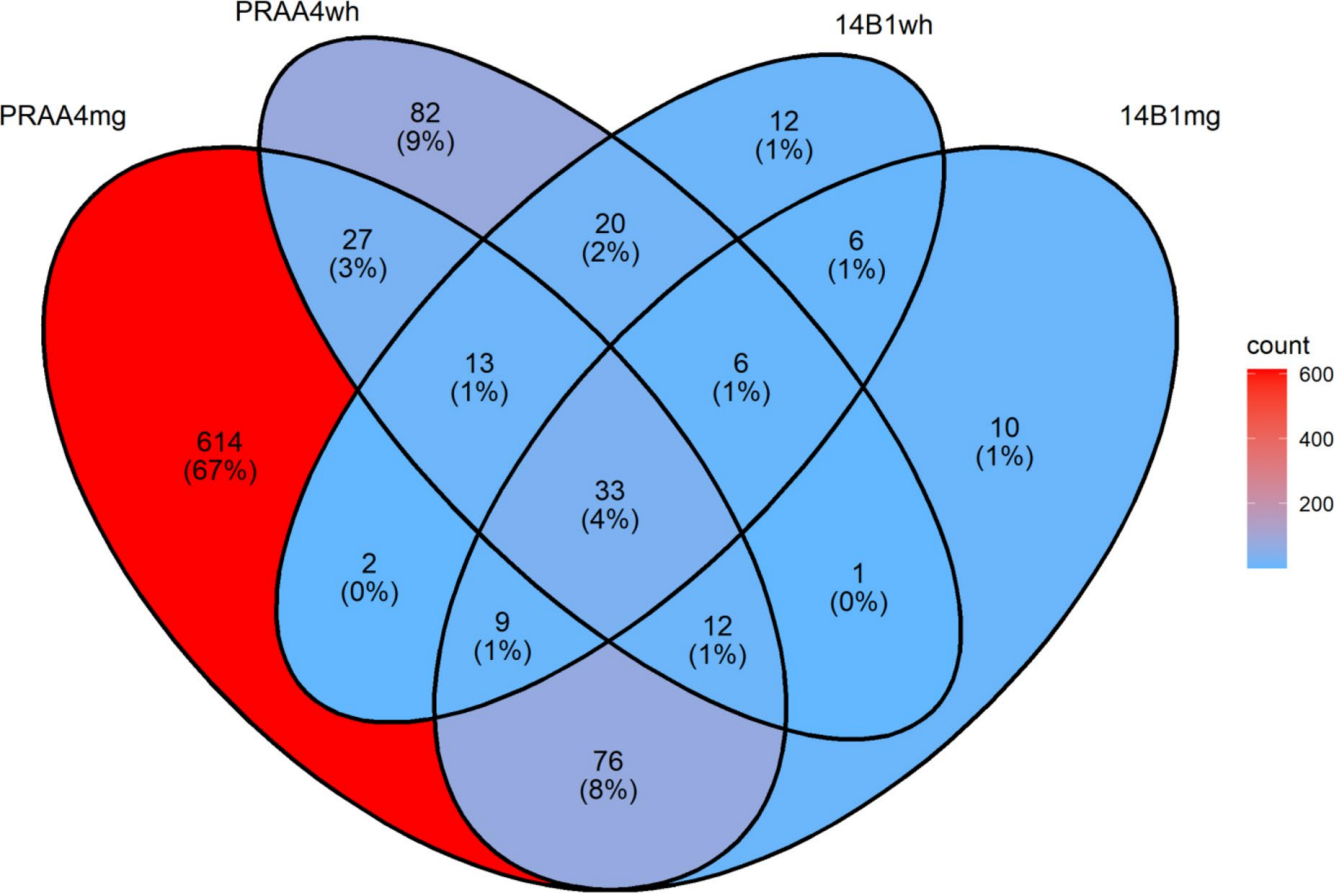
Venn diagram depicting counts of shared (or unique) differentially expressed genes (DEGs) among the four treated samples contemplated in this study. Note that whether a DEG was up- or down-regulated was not considered for the amounts reported here—that is, a DEG up-regulated in one context and down-regulated in another counted as a single DEG. Indeed, only five of the 923 DEGs exhibited this mixed-direction regulation, and these are presented in detail on the worksheet “mixed-case_DEGs” provided in the supplementary file, “VennDEGsupport.SupplementaryMaterial.xlsx”.

Differential gene expression analysis yielded the following overall DEG counts: 14B-1 midgut vs. control midgut = 153 DEGs (88 up, 65 down), 14B-1 whole-insect vs. control whole-insect = 101 DEGs (69 up, 32 down), PRAA4-1 midgut vs. control midgut = 786 DEGs (239 up, 547 down), and PRAA4-1 whole-insect vs. control whole-insect = 194 DEGs (101 up, 93 down). Gene-level, sample-specific expression estimates, as well as a listing of DEG-associated isoforms for which hits to reference proteins were observed, are provided for these comparisons in the supplemental file on worksheets labeled “res_14B1mg”, “res_14B1wh”, “res_PRAA4mg” and “res_PRAA4wh”, respectively. A comprehensive listing of all DEGs is presented on the worksheet labeled “gene_contrasts_with_TPM” of the supplemental file. A Venn diagram summarizing counts of DEGs shared among the four treated sample types is presented in Fig. 1. Tables detailing DEGs contained in each of the regions of overlap in the diagram are presented in the supplementary file, “VennDEGsupport.SupplementaryMaterial.xlsx”.

Of the transcripts expressed from the nonredundant set of DEGs identified above (there were 2,897 such mRNA sequences; see the supplemental file’s “DEG-associated_isoforms” sheet), a total of 2,331 (80.46%) had a hit with the NCBI NR database, 1,773 (61.20%) exhibited one or more hits to reference Pfam protein domain families and 683 of these (38.52%) had one or more associated GO terms. This annotation information, as well as transcript-level expression data, is provided on the supplemental workbook’s “DEG-associated_isoforms” sheet.

Bacterial genomes of *C. subtsugae* and *C. sphagni* were analyzed for BGCs. The PRAA4-1 genome had 18 identified BGCs, of which twelve matched similar clusters in the database and only three exhibited a match exceeding 50% similarity. The 14B-1 genome had 14 total BGCs, nine of which matched to similar clusters in the database and, of these, five had similarity over 50%. The full list of identified clusters is presented in Table 2.

**Table 2:** Percent similarity of annotated biosynthetic gene clusters identified in published genomes of *C. subtsugae* PRAA4-1^T^ and *C. sphagni* 14B-1^T^.

Type	Most similar known cluster	Chromobacterium subtsugae	Chromobacterium sphagni
T3PKS	2,4-diacetylphloroglucinol	**75%**	n.d.
arylpolyene	aryl polyenes	20%	44%
NRP-metallophore, NRPS	chromobactin	n.d.	**100%**
transAT-PKS-like	difficidin	33%	n.d.
transAT-PKS-like	etnangien	18%	n.d.
transAT-PKS-like	etnangien	37%	n.d.
betalactone	fengycin	13%	13%
hydrogen-cyanide	hydrogen cyanide^a^	**100%**	**100%**
T3PKS	lagriene	21%	n.d.
transAT-PKS	lagriene	17%	n.d.
NRPS	sulfazecin	n.d.	**52%**
terpene	surfactin	8%	8%
NRPS	viobactin	30%	**92%**
indole	Violacein^a^	**100%**	**100%**
hglE-KS, NRPS, T1PKS	xenocoumacin I/ xenocoumacin II	n.d.	14%

Clusters identified using the program bacterial antiSMASH (version 7.1.0). “n.d.” ~ no data. Bold indicates clusters with > 50% identity match.

^a^Compounds previously associated with *Chromobacterium *insecticidal activity^8,12^. Note that two distinct transAT-PKS-like BCGs similar to an etnangien cluster were observed.

## Discussion

*C. subtsugae* strain PRAA4-1^T^ exhibits oral toxicity against a broad range of important agricultural insect pests, spanning at least dipterans, coleopterans, lepidopterans, some hemipterans and tetranychidans. In contrast, *C. sphagni* 14B-1^T^ exhibits similar oral toxicity to that of *C. subtsugae *against lepidopterans, but not dipteran or coleopteran insects^6^. The degree to which *C. sphagni* shares the machinery used by *C. subtsugae *to achieve its own insecticidal phenotype is not known. Indeed, the actual molecular mechanisms utilized by either of these species remain poorly characterized, although they appear distinct from the crystal toxin-based system characteristic of most insecticidal strains of Bt^33^. Studying molecular-level larval responses to feeding on various *Chromobacterium* species should help provide indirect insights into how these bacteria achieve their toxicity.

Some DEGs identified using the transcriptome sequence resources generated by this study were either novel (i.e., no NR hits) or had hits against proteins of uncharacterized function. Future functional genetics work will be needed to understand the molecular roles of these genes. Nevertheless, differential gene analysis did reveal a subset of genes with known functions, which can provide insight into both the mechanisms of toxicity and host immune responses triggered by the bacteria (see Table 3 for representative cases). A DEG common to all treatments and tissue types was TRINITY_GG_15562_c0_g1, whose transcripts encode for attacin, an antimicrobial peptide^34^ (see supplementary file, “VennDEGsupport.SupplementaryMaterial.xlsx”, for this gene and all others discussed below). In addition to this gene, there were several DEGs encoding attacin observed in other subgroupings: the inferred (and presumably alternatively spliced) gene, TRINITY_GG_15562_c0_g2, which was up-regulated in whole-tissue (wh) samples from both treatments; TRINITY_GG_10166_c0_g1, up-regulated in wh, *C. subtsugae*-treated samples; and TRINITY_GG_15562_c6_g4, also up-regulated in the wh samples following *C. subtsugae* treatment. This suggests that there may be differences in splice or gene variants that could make attacin more effective in these various biological contexts. More dedicated analysis will be required to learn how these variants could be selectively utilized by the cell.

**Table 3:** Table of select DEGs and the treatment types in which they were identified as being differentially expressed.

Gene	Inferred functional role	PRAA4-1^T^		14B-1^T^	
		Midgut	Whole	Midgut	Whole
TRINITY_GG_15562_c0_g1	attacin	X	X	X	X
TRINITY_GG_15562_c0_g2	attacin		X		X
TRINITY_GG_10166_c0_g1	attacin		X		
TRINITY_GG_15562_c6_g4	attacin		X		
TRINITY_GG_21289_c0_g1	cecropin	X	X		
TRINITY_GG_13730_c0_g1	moricin		X		X
TRINITY_GG_301_c76_g1	fatty acid-binding protein 2			X	
TRINITY_GG_16749_c18_g1_i1	glucose 1-dehydrogenase-like protein				X
TRINITY_GG_10534_c133_g2	chymotrypsin-2-like isoform X1	X			
TRINITY_GG_1847_c1_g1	cytochrome P450	X	X	X	X
TRINITY_GG_15158_c0_g1	cytochrome P450	X	X	X	X
TRINITY_GG_8242_c0_g1	zinc transporter	X		X	
TRINITY_GG_8034_c4_g2	zinc transporter	X		X	
TRINITY_GG_21328_c32_g1	gelsolin			X	X

The first column is the name of the gene in the global assembly. The second column is the gene’s most likely functional role based on matches of its encoded transcripts to the NR database. The final four columns correspond to each treatment type, with an “X” indicating that a gene was identified as being differentially expressed in that comparison (relative to its respective negative control as described in Materials and Methods).

There were several other similar genes that were up-regulated in other comparisons, including TRINITY_GG_21289_c0_g1 (one of whose transcripts encoded cecropin, an antimicrobial peptide^35^), which was exclusively found to be differentially expressed (DE) in both PRAA4-treated tissue types, as well as TRINITY_GG_13730_c0_g1 (encoding for moricin, an antibacterial peptide^36^), which was found to be DE in wh samples from both bacterial treatments.

Aside from genes involved in the synthesis of antibacterial and antimicrobial peptides, genes involved in other defense pathways were up-regulated. For instance, TRINITY_GG_301_c76_g1, encoding fatty acid-binding protein 2, a molecule involved in lipid metabolism, was found exclusively in the *C. sphagni *midgut (mg) treatment group. Lipids have a crucial role in many aspects of insect immunity^37^; thus, increased lipid metabolism could suggest these pathways are up-regulated in general. Fatty acid binding protein 2 was up-regulated in *Spodoptera litura *in response to starvation^38^, and in the current study may be the result of a behavioral response to intoxication.

The gene, TRINITY_GG_16749_c18_g1_i1, comprising at least one transcript for the glucose 1-dehydrogenase-like protein, was found to be DE only in the wh sample under *C. sphagni* treatment. Work in *Mamestra brassicae *larvae suggests that glucose dehydrogenase activity is associated with higher rates of encapsulation, which is a common insect immune response^39^.

Among DEGs exclusive to the *C. subtsugae *mg group, an up-regulated gene, TRINITY_GG_10534_c133_g2, encoded a transcript corresponding to chymotrypsin-2-like isoform X1. Chymotrypsin is a serine protease which can be involved in the proPR activating pathway, which ultimately produces melanin as well as several other molecules utilized in the immune response^40,41^.

There were also a few notable DEGs that were not necessarily involved in large immune system pathways. Among DEGs common to all treatments and tissue types were two up-regulated genes encoding cytochrome P450 enzymes: TRINITY_GG_1847_c1_g1 and TRINITY_GG_15158_c0_g1. Members of this gene family are often involved in xenobiotic detoxification^42–44^, and thus it may be that they play a role in breaking down toxic metabolites produced by the bacteria. Alternatively, P450 enzymes may be involved in catalyzing the production of epoxyoctadecamonoenoic acids, which have a role in insect immunity^45^. A P450 enzyme was up-regulated in the midgut of the eastern spruce budworm, *Choristoneura fumiferana*, in response to ingestion of a sublethal dose of Bt’s Cry1Ab toxin^46^.

Two genes encoding zinc transporters, TRINITY_GG_8242_c0_g1 and TRINITY_GG_8034_c4_g2, were found to be DE only in mg samples from both bacterial treatments, contexts in which they were down-regulated. Zinc is an essential trace metal in many different biological contexts, including the immune system, and can be involved in processes such as enhancing antimicrobial peptides^47^. Additionally, intracellular decreases in zinc levels can induce apoptosis via caspase activation^48^. Further, both *C. subtsugae* and *C. sphagni *genomes contain multiple metalloprotease genes, which often require zinc, and may contribute to insecticidal activity as shown in other insect-microbe interactions^47,49^. Therefore, the insects may downregulate these transporters to sequester zinc and reduce availability for the bacteria.

Both wh and mg tissue samples under *C. sphagni *treatments had up-regulation of a gene encoding gelsolin, TRINITY_GG_21328_c32_g1, a molecule involved in organizing actin^50^. Actin has a key role in a myriad of cellular processes, including phagocytosis^51^, and it is possible that up-regulation of gelsolin could be indicative of an increase in phagocytotic activity so as to deactivate and eliminate invading pathogenic bacteria. Up-regulation of gelsolin may also indicate the occurrence of cytoskeletal rearrangements of midgut cells common to a number of pathological processes, such as exposure to pesticidal proteins of Bt or *Photorhabdus luminescens*, and gelsolin was found to be up-regulated in the midgut of eastern spruce budworm that were fed Cry1Ab^46^.

**Fig. 2:**
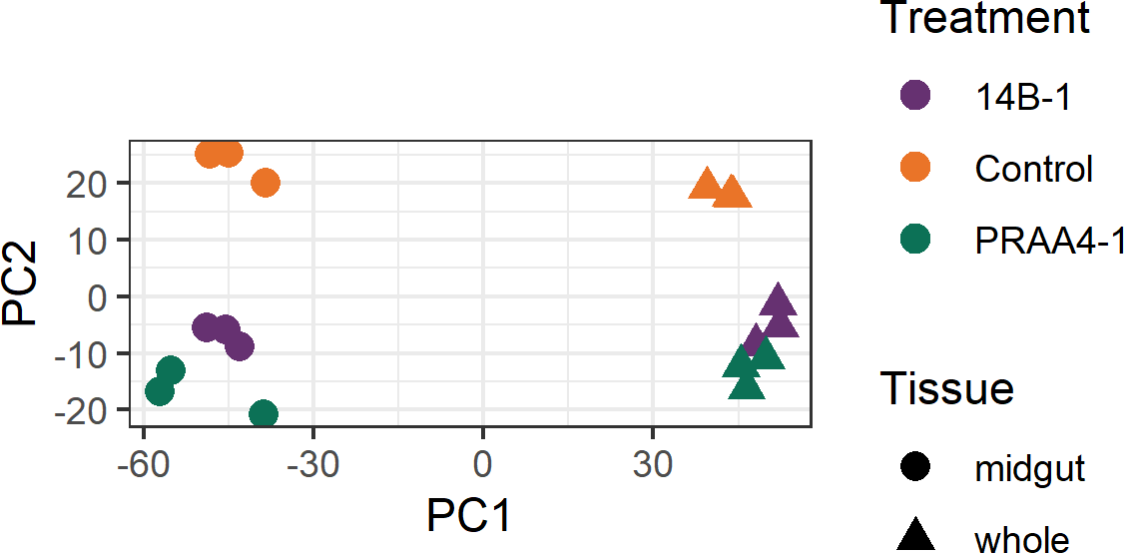
Principal component analysis of gene expression among all 18 RNA-Seq samples. Shape indicates tissue type (“midgut” ~ midgut-only samples and “whole” ~ whole insect samples). Color indicates treatment group (“Control” ~ negative control, “14B-1” ~ *C. sphagni* strain 14B-1^T^ and “PRAA4-1” ~ *C. subtsugae* strain PRAA4-1^T^). Note that two of the whole-insect control sample points are essentially superimposed on one another and visually indistinguishable.

Somewhat counterintuitively, a larger number of DEGs was observed in midgut samples relative to whole-insect samples (which comprised midgut tissue), for both bacterial treatments:

153 DEGs overall for 14B-1 mg vs. control mg > 101 DEGs for 14B-1 wh vs. control wh, and 786 DEGs for PRAA4-1 mg vs. control mg > 194 DEGs for PRAA4-1 wh vs. control wh. Specific causes for this are not clear, though there is likely a higher amount of “noise” in the whole-insect samples arising from genes expressed in tissues not involved in infection response. This noise may prevent midgut-expressed genes with more subtle expression differences from being detected in whole-insect samples: with a greater number of genes expressed in whole insect rather than midguts, such a discrepancy in raw counts could affect gene count normalization, gene-wise dispersion and shrinkage results calculated by DESeq2^18^. Other potential factors may include ordinary biological variation among the insects included in each biological sample and variation in sequencing volume achieved for the various replicates (see Table 1). More consistent library sequencing depths than achieved in this study by the sequencing vendor (see Table 1) would have been preferable, yet this is unlikely to have had an appreciable effect on the results. A principal component analysis on the unassembled reads demonstrated that samples from a given treatment and/or tissue type cluster together unambiguously (see Fig. 2). The computational methods utilized in this work are also inherently robust to differences in sequencing depth—for instance, the Trinity package incorporates in silico normalization functions for use in assembly and DESeq2 package accounts for such variability via its normalization/ size factor calculations.

No attempts were made to experimentally deplete prokaryotic RNAs collected from midgut or whole-insect specimens prior to sequencing library construction. However, as prokaryotic mRNA molecules lack the substantive polyadenylation exhibited by eukaryotic transcripts, they likely experienced degradation before Illumina sequencing could capture them. Note also that the transcriptome assembly utilized the genome-guided setting in Trinity, and so only transcripts mapping to the reference spongy moth genome were generally included in the final transcriptome assembly, effectively filtering out RNA from non-target sources (e.g., gut bacteria). Lastly, transcripts were annotated by comparison with the NR, Pfam and GO databases, and these correspondences may also help to limit the risk that genes under consideration are of prokaryotic origin rather than the eukaryotic host, *L. dispar*.

To provide insight into insecticidal activity of both *Chromobacterium* strains, the available genomes were investigated. Surprisingly, each strain had a high similarity match in antiSMASH with only a few BGCs. Both *C. subtsugae* and *C. sphagni* have complete hydrogen cyanide and violacein BGCs, which are known insecticidal factors associated with *C. subtsugae*^7,9^. Two siderophore BGCs characterized in *C. violaceum*, chromobactin and viobactin, were identified in *C. sphagni*, which may contribute to insecticidal activity as previous insect pathosystems have demonstrated^47,52^.

In addition to BGCs, other genes and gene families were identified as potential contributors to insecticidal activity^9^. Both *C. subtsugae* and *C. sphagni *genomes contain numerous annotated, yet distinct, protease genes. Many studies have demonstrated proteases to be associated with insecticidal activity or antimicrobial peptide interference^49,53^. Two putative chitinases produced by *C. subtsugae *PRAA4-1 known to have insecticidal activity in corn rootworm^10^ were not encoded in the *C. sphagni* 14B-1 genome. Furthermore, it is not yet known whether *C. sphagni* shares the capacity to produce “chromamide A” or the “uncharacterized dipeptide” reported as toxic factors produced by *C. subtsugae*^8^. These unique factors likely play a role to the broader toxicity range of *C. subtsugae* compared to more narrow *C. sphagni*, and may contribute to the *L. dispar* DEGs between the two inoculated species. Further experimental evidence could validate the impact of these genes and their products in *Chromobacterium*-insect interactions in *L. dispar* and across orders. Although this study focused on the genome of the *C. subtsugae* type strain, PRAA4-1 (the strain with which *L. dispar* insects were inoculated), genomes of other *C. subtsugae *strains have been published (e.g., ATCC 31532 and ΔvioS^54^) and could be investigated in the future.

Independent ingestion and infection in spongy moth larvae of two distinct *Chromobacterium* species was shown to trigger several differential immune responses to the focal bacteria. Future work within additional lepidopteran species will be needed both to better understand insect responses to *Chromobacterium* infection generally, as well as to more completely characterize differences in lepidopteran response to each insecticidal species. For instance, zinc seems to be important in the response to infection by both species, but it is unclear what its exact cellular role is. There are also notable differences in insect response to infection by the two species with different complements of antimicrobial peptide coding genes up-regulated for each. Further work will be needed to identify and correlate key biological differences between the two bacterial species with targeted insect responses, which in turn will improve biopesticide strain selections.

## Electronic supplementary material

Below is the link to the electronic supplementary material.

Supplementary Material 1

Supplementary Material 2

## Data Availability

Sequencing data described in this report are publicly available from the NCBI under the BioProject accession number PRJNA1141131. All other data generated and/or analyzed in this study are otherwise included in this published article and its Supplementary Files. Data are also available from the corresponding author upon request.
